# CD57-Expressing Lymphocytes: From Chronic Viral Response to Age-Related Inflammation

**DOI:** 10.3390/cells15050403

**Published:** 2026-02-26

**Authors:** Isabel María Vallejo-Bermúdez, Mabel Rocio Miranda-Echagüe, Silvia Fernández-Álvarez, Irene Reina-Alfonso, Laura Blanca-Pariente, Alexander Batista-Duharte, Alejandra Pera

**Affiliations:** 1 Immunology and Allergy Group (GC01), Maimonides Biomedical Research Institute of Cordoba (IMIBIC), University of Cordoba, Reina Sofia University Hospital, Av. Menendez Pidal s/n, 14004 Cordoba, Spain; b82vabei@uco.es (I.M.V.-B.); m.mirandaechague@student.unisi.it (M.R.M.-E.); z02reale@uco.es (I.R.-A.); z52blpal@uco.es (L.B.-P.); 2 Department of Cell Biology, Physiology and Immunology, University of Cordoba, Av. Menendez Pidal s/n, 14004 Cordoba, Spain; b82feals@uco.es

**Keywords:** CD57^+^ lymphocytes, immunosenescence, cytomegalovirus (CMV), T cells, NK cells, terminal differentiation

## Abstract

**Highlights:**

**What are the main findings?**

- CD57 marks advanced differentiation across T and NK cells, but CD57+ CD4+ and CD57+ CD8+ T cells show distinct developmental trajectories and functional programs rather than a uniform senescent state.
- CMV-driven chronic stimulation is the dominant force shaping CD57^+^ lymphocyte expansion, linking viral persistence with cytotoxic specialization and immune remodelling.

**What are the implications of the main findings?**

- CD57 should be interpreted as a context-dependent differentiation and immune-history marker—not a direct therapeutic target—and CD57+ CD4+ and CD57+ CD8+ subsets must be analyzed separately.
- Proper interpretation of CD57^+^ subsets, with CMV stratification, improves immune profiling in aging, chronic infection, cardiovascular and autoimmune disease, cancer, and vaccine-response studies.

**Abstract:**

CD57-expressing lymphocytes constitute a distinct subset of immune cells with enhanced cytotoxic and pro-inflammatory functions. Initially described in the context of chronic viral infections, most notably cytomegalovirus (CMV), these cells are now recognized as central contributors to immunosenescence and age-related immune dysregulation. Their progressive accumulation reflects prolonged antigenic exposure and sustained immune activation, thereby linking persistent viral infections with long-term disruptions of immune homeostasis. Emerging evidence indicates that CD57 expression denotes a state of terminal differentiation in both T and natural killer (NK) cell compartments, and is associated with cytotoxicity, altered cytokine secretion, and a pro-inflammatory phenotype. This review summarizes the phenotypic and functional characteristics of CD57^+^ lymphocytes, examines their association with CMV and other chronic viral infections, and explores their potential role in ageing and age-related diseases. Elucidating the biology of CD57^+^ lymphocytes in the context of chronic viral infections may provide novel insights into immune ageing and help identify potential targets for therapeutic strategies aimed at restoring immune balance in older adults.

## 1. Introduction

Age-related changes in the immune system reduce its ability to respond to new challenges. This condition, known as immunosenescence, is associated with multiple functional alterations of the immune system, including (i) increased susceptibility to infections, (ii) impaired immunosurveillance with a consequent higher risk of cancer development, (iii) reduced responsiveness to vaccination, (iv) compromised memory T-cell responses, (v) greater propensity to autoimmune disorders, and (vi) decreased telomerase activity in T cells [1,2]

In a longitudinal study of nonagenarian and octogenarian Swedish individuals, it was established that an Immune Risk Profile (IRP) is determined by a variety of immunological parameters associated with lifespan, predicting mortality and morbidity [3,4,5]. The primary feature of this IRP was a reduction in CD8^+^ naïve T cells and an accumulation of memory CD8^+^ T cells. Another feature was the increase in the expression of CD57 in these cells, together with the loss of CD28 and CD27 markers. These changes in the T-cell repertoire are associated with thymic involution beginning at puberty, which leads to a progressive decline in the output and replacement of naïve T cells. In addition, chronic immune stimulation by persistent pathogens, particularly cytomegalovirus (CMV) seropositivity, promotes oligoclonal expansions, narrowing the T-cell repertoire and reducing the pool of cells capable of responding to novel antigens [4,6]. CMV is therefore considered one of the main drivers accelerating immunosenescence [7,8].

CMV is a common beta-herpes virus that, after primoinfection, remains latent evading the immune system surveillance [9]. The global average seroprevalence ranges from 45 to 100%, which increases with age and is related to geographic location, ethnicity, socioeconomic status, and education [9,10,11]. In this sense, South America, Asia, and Africa show higher CMV prevalence than the USA and northern European countries such as Germany and England [12]. In CMV-seropositive young individuals, a high frequency of polyfunctional CD57^+^ T cells has been observed, suggesting that this subpopulation may enhance immune response and infection control [13]. However, in older individuals, these CMV-associated polyfunctional cells may also promote chronic inflammation, thereby impairing optimal immune responses [14].

Both CD57+ CD8+ and CD57+ CD4+ T cells have been linked to several age-associated pathologies. For example, elevated frequencies of CD57+ CD4+ T cells have been detected in patients with acute heart failure and correlate with adverse clinical outcomes [15,16]. Likewise, the expansion of CD57+ CD8+ T cells has been associated with increased arterial stiffness and heightened cardiovascular risk [17]. Beyond the T cell compartment, CD57^+^ natural killer (NK) cells, often expanded following CMV infection, have also been implicated in chronic inflammation, autoimmune disorders, and cancer [18,19,20]. Thus, the accumulation of these pro-inflammatory lymphocytes during ageing, particularly in CMV-positive individuals, suggests a dual role: while potentially protective during acute infection, they may drive “inflammaging” and contribute to age-related pathologies. Finally, recent evidence reinforces this concept, showing that CMV-seropositive individuals who have experienced SARS-CoV-2 infection display accelerated T-cell immunosenescence, characterized by an expansion of highly differentiated CD57^+^ T cells and a reduced capacity for immune restoration [11]. Collectively, these findings underscore CD57 expression as a key functional marker linking viral persistence, immune senescence, and the development of chronic age-related diseases.

This review provides an integrated overview of CD57-expressing lymphocytes, emphasizing their central position at the intersection of viral persistence, immune ageing, and chronic inflammation. Understanding how these cells shift from protective immunity to pathogenic contributors offers key insights into the mechanisms linking CMV infection, immunosenescence, and the emergence of age-related inflammatory and degenerative diseases.

### Literature Search Strategy

This article is a narrative review and was not designed as a formal systematic review or meta-analysis. The literature discussed was identified primarily through structured searches in PubMed/MEDLINE, complemented by reference-list screening of relevant articles. Search terms included combinations of “CD57”, “HNK-1”, “Leu-7”, “T cells”, “CD4”, “CD8”, “NK cells”, “immunosenescence”, “cytomegalovirus”, “CMV”, “chronic infection”, and “terminal differentiation”. Priority was given to peer-reviewed original studies and high-quality reviews published in English, with particular emphasis on mechanistic, phenotypic, and clinically oriented immunology studies. Study selection was based on relevance to CD57 biology, differentiation status, and clinical associations. No formal systematic quality scoring or meta-analytic methods were applied, consistent with the narrative scope of this review.

## 2. Biology of CD57 Marker

The CD57 antigen (also known as HNK-1 or Leu-7) is a carbohydrate epitope first identified in 1981 by Abo et al. on human natural killer (NK) cells [21], and later found on subsets of T lymphocytes and neural cells. Structurally, CD57 corresponds to a unique sulphated trisaccharide 3-O-sulfated glucuronic acid (GlcAβ1-3Galβ1-4GlcNAc-R) located at the non-reducing terminus of glycan chains [22]. This glycoepitope is synthesized through the sequential action of β1,3-glucuronyltransferases (B3GAT1/GlcAT-P and B3GAT2/GlcAT-S), which add glucuronic acid to galactose residues, followed by the HNK-1 sulfotransferase (CHST10), which catalyzes sulfation at the C-3 position of GlcA [23]. The sulphate group is critical for the adhesive properties of the molecule [24]. This terminal sulfation step is essential for the biological activity of the HNK-1 epitope, which decorates multiple glycoproteins and glycolipids in both neural and immune tissues [25]. The expression of CD57, therefore, depends not on a single protein, but on the coordinated regulation of glycosyltransferases and sulfotransferases within the Golgi apparatus [26]. A recent study has shown that small extracellular vesicles (sEVs) can mediate the direct transfer of HNK-1 and its major biosynthetic enzyme, GlcAT-P, between cells, highlighting a non-genetic pathway of glycan remodelling through intercellular communication [27].

In the nervous system, the HNK-1 epitope is abundantly expressed on glycoproteins such as myelin-associated glycoprotein (MAG) [28], neural cell adhesion molecule (NCAM) [29], the mammalian glycoproteins PM/Po [30] and tenascin-R [31], where it participates in cell–cell and cell–matrix adhesion, neurite outgrowth, and synaptic plasticity [32,33]. Expression of the HNK-1 epitope in non-neural tissues indicates a broader role in modulating cell–matrix interactions and tissue architecture. In the immune system, CD57 expression constitutes a complex and multifaceted marker that reflects an individual’s cumulative immune history, particularly in the context of persistent infections such as CMV [8]. It is generally accepted that CD57 expression identifies terminally differentiated or senescent lymphocytes, particularly T and NK cells, which exhibit enhanced cytotoxic capacity but limited proliferative potential [18,34,35]. However, in a recent report, [36] analyzed CD57 expression on CD4^+^ and CD8^+^ T cells in two cohorts of healthy adults, considering age, CMV serostatus, and cellular differentiation. They combined phenotypic analysis with functional assays for proliferation and senescence-associated β-galactosidase activity. Their findings demonstrated that CD57 expression was strongly associated with CMV seropositivity and T cell differentiation, but not with chronological age. Consequently, they concluded that CD57 should not be considered a strict marker of T cell senescence, but rather of immunological age, reflecting cumulative immune stimulation and viral exposure history.

In summary, CD57 is not only a surface marker but also a conserved molecular motif involved in cell recognition and adhesion across biological systems. It links neural interaction mechanisms with immune maturation and ageing and is strongly associated with chronic viral infection, inflammation, and age-related immune remodelling. Its expression reliably identifies terminally differentiated, highly cytotoxic T-cell subsets, highlighting its value as a functional marker in the study of immune regulation and immunosenescence [8,35,37]. In the following section, we will analyze the relevance of CD57 expression across different lymphocyte populations, exploring its functional implications in immune regulation and ageing.

## 3. CD57 in αβ T Cells

CD57 expression in T lymphocytes is generally associated with advanced differentiation and is frequently accompanied by the loss of the co-stimulatory receptor CD28. In both CD4^+^ and CD8^+^ compartments, CD57^+^ T cells tend to display a CD28^−^ (CD28null) phenotype, reflecting cumulative antigenic stimulation and progressive differentiation along the effector pathway. The CD28nullCD57^+^ T-cell profile has been consistently linked to enhanced cytotoxic potential and distinctive transcriptional programmes, although its precise biological meaning remains debated [35,38,39].

CD28 is a costimulatory receptor expressed on naïve CD4^+^ and CD8^+^ T cells that binds B7-1 (CD80) and B7-2 (CD86) on antigen-presenting cells. This interaction triggers signalling pathways that promote cytokine production, T-cell proliferation, survival, and differentiation through mediators such as Akt, NF-κB, and mTOR [40]. The decline in CD28 expression on both CD4^+^ and CD8^+^ T lymphocytes is a well-recognized feature of immunosenescence [41]. Nonetheless, it is important to distinguish transient downregulation of CD28 from the permanent loss of this molecule, as only the latter reflects long-term changes associated with ageing [42]. Early models of CD8^+^ T cell differentiation suggested a linear trajectory in which activated CD8^+^ T cells progressively downregulate CD28 and subsequently acquire CD57 expression. However, accumulating evidence indicates that this process is more complex, with CD28 and CD57 defining multiple CD8^+^ T-cell subsets characterized by distinct phenotypic, functional and transcriptional profiles. These observations challenge the simplistic view of CD57 as a mere marker of senescence and underscore its role in shaping CD8^+^ T-cell heterogeneity [43]. Experimental observations further support this non-linear model of CD8^+^ T cells differentiation, as both CD28nullCD57^−^ and CD28^+^CD57^+^ CD8^+^ T cells can be detected in peripheral blood [44,45,46] questioning the traditional view of mutually exclusive expression of these markers.

The co-stimulatory receptors CD28 and CD27 are widely used to define the differentiation status of CD8^+^ T cells. In general, early-differentiated CD8^+^ T cells retain both markers (CD28^+^CD27^+^), whereas more differentiated populations progressively lose one or both molecules, resulting in multiple phenotypic combinations that reflect functional and maturational heterogeneity [47,48]. Unlike CD4^+^ T cells, in which the loss of CD27 and CD28 follows a more sequential pattern, CD8^+^ T cells display all possible CD27/CD28 expression profiles, indicating that their differentiation does not follow a strictly linear trajectory [47]. Within this framework, CD8^+^ T cells can be broadly categorized into early (CD28^+^CD27^+^), intermediate (CD28^−^CD27^+^ or CD28^+^CD27^−^), and late (CD28^−^CD27^−^) differentiation stages [47,48].

### 3.1. CD8^+^ T Cells

CD57 expression is commonly associated with advanced differentiation and replicative senescence in CD8^+^ T cells. Although CD57+ CD8+ T cells are predominantly enriched in the CD28^−^CD27^−^ phenotype, this subset is phenotypically heterogeneous, and a proportion of CD57^+^ cells retain CD27 and/or CD28 expression [45]. Consistent with their late differentiation status, CD28^−^CD27^−^CD57^+^ CD8^+^ T cells exhibit markedly reduced telomerase activity and features of replicative senescence, supporting their classification as terminally differentiated effector populations [44,49]. In this way, it has been reported that downregulation of costimulatory molecules CD28 and CD27 in terminally differentiated effector memory T cells (TEMRA, CCR7-CD45RA+) can be mediated via epigenetic regulation [50].

CD57+ CD8+ T cells frequently express CD94/NKG2A, inhibitory receptors typically associated with NK cells (iNKRs), which may modulate their activation threshold and survival [51,52].

Within this population, CD27^−^CD57^+^ CD8^+^ T cells have been proposed as the most senescent subset, characterized by resistance to apoptosis [43,44,45,46,47,48,49,50,51,52,53,54,55,56]. This impaired apoptotic response could explain their progressive accumulation in older individuals, contributing to the phenomenon of immunosenescence. Nevertheless, it has been shown that CD57+ CD8+ T cells can still proliferate in response to persistent antigenic stimulation and, in some cases, reactivate telomerase to overcome cell-cycle arrest, indicating that not all are terminally senescent [54]. This dynamic interplay between CD28 and CD57 underscores the plasticity of CD8^+^ T cell responses and their role in immune surveillance.

Heterogeneous functional capacities have been reported within the CD57+ CD8+ T cell population. Upon activation, these cells exhibit enhanced cytotoxic activity [55], characterized by the expression of perforin, granzyme B, and granulysin [56].

In addition, CD57+ CD8+ T cells produce high levels of pro-inflammatory cytokines such as IFN-γ and TNF-α [13,57], and, interestingly, IL-5 [54]. These cytokines may also contribute to the maintenance and expansion of highly differentiated CD57^+^ subsets, as inflammatory signals, particularly IFN-γ induce IL-15 expression and trans-presentation by myeloid and tissue cells, and IL-15 is a well-established driver of survival and proliferation in terminally differentiated and CD28null/CD57^+^ CD8^+^ T cells [57,58,59]. Moreover, TNF-α has been shown to promote CD28 downregulation, further biassing the pool toward highly differentiated CD28null phenotypes that frequently co-express CD57 [60]. Consequently, their cytotoxic functions enable the recognition and elimination of aberrant cells, while they may also contribute to the exacerbation of inflammatory processes.

Transient FOXP3 expression during CD8^+^ T-cell activation may function to restrain excessive immune activation and tissue damage at inflammatory sites [61,62]. Within the CD8^+^ compartment, regulatory T-cell populations (CD8^+^ Tregs) are phenotypically heterogeneous and can be partly distinguished by CD57 expression, which reflects differentiation status and functional capacity. CD8^+^ regulatory activity has been most consistently described in CD28null subsets that exert MHC class I–restricted suppression of CD4^+^ T cells and antigen-presenting cells; these populations are commonly CD57^−^ or phenotypically mixed and retain measurable proliferative potential [61,63]. By contrast, CD57+ CD8+ T cells are predominantly highly differentiated effectors with strong cytotoxic and pro-inflammatory profiles and limited proliferative capacity. Although they can produce regulatory cytokines such as IL-10 under specific conditions, including chronic viral infection, they do not represent a stable FOXP3^+^ regulatory lineage [43]. Moreover, FOXP3 expression in CD8^+^ T cells can be transiently induced by persistent antigenic stimulation without conferring durable regulatory identity and is not consistently linked to CD57 expression [63].

Further evidence of the functional heterogeneity of CD8^+^ T-cell subsets defined by CD28 and CD57 expression comes from transcriptomic and phenotypic analyses in young and elderly individuals. Onyema et al. showed that ageing is associated not only with an expansion of CD28nullCD57^+^ CD8^+^ T cells but also with an increased frequency of CD28^+^CD57^+^ CD8^+^ T cells. These subsets displayed distinct patterns of senescence- and apoptosis-related markers, with the highest expression of p16 and p21, and elevated levels of Bcl-2 and CD95, in the CD28^+^CD57^+^ subset. Moreover, differentiation and homing markers were unevenly distributed among subsets: CD62L and CCR7 were predominantly expressed in CD28^+^CD57^−^ CD8^+^ T cells, whereas CD28nullCD57^+^ and CD28^+^CD57^+^ cells exhibited low and intermediate levels, respectively. In addition, PD-1 and CD45RO were most highly expressed in CD28^+^CD57^+^ cells, while CXCR2 expression was preferentially associated with CD28nullCD57^+^ subset. Altogether, these findings indicate that CD8^+^ T-cell subsets defined by CD28 and CD57 expression are characterized by distinct molecular and functional profiles, supporting the concept that CD57+ CD8+ T cells constitute a heterogeneous population rather than a uniform senescent compartment [45,46]. CD57+ CD8+ subsets may also display variable expression of checkpoint and inhibitory receptors, including PD-1, whose levels are determined by antigenic and inflammatory conditions rather than representing an intrinsic feature uniformly associated with CD57 expression [64,65,66].

In human disease contexts, such as chronic infections and cancer, CD57+ CD8+ T cells exhibit hallmarks of advanced differentiation, including diminished proliferative capacity and altered expression of survival factors such as Bcl-2, consistent with extensive replicative history and exhaustion-like phenotypes [50]. In the context of chronic inflammation, FOXP3 expression can be transiently induced in CD8^+^ T cells [63,67] and in CD57+ CD8+ T cells [68]. However, these populations are functionally distinct. Specifically, in a study of patients with stage IV gastric carcinoma undergoing treatment with activated autologous lymphocytes, Akagi et al. [68] demonstrated that FOXP3 expression in CD57+ CD8+ T cells was transient and occurred in the absence of associated regulatory function during the natural course of CD8^+^ T-cell differentiation. In contrast, CD57^−^FOXP3^+^ CD8^+^ T cells were appropriately classified as CD8^+^ regulatory T cells. These findings highlight that CD57 expression defines functionally heterogeneous populations within the CD8^+^ T-cell compartment, encompassing both pro-inflammatory and regulatory subsets.

Within the conventional CD8^+^ T-cell compartment, NKT-like T cells, expressing the CD56 marker, represent a differentiated subset. Phenotypically, these cells typically display an effector-memory/terminal-effector profile, enriched cytotoxic machinery, and rapid effector responsiveness, and they are known to expand with ageing and chronic viral infection, particularly CMV [69,70,71]. CD57^+^ NKT-like CD8^+^ T cells are preferentially expanded in CMV-seropositive individuals and show features of late differentiation, commonly associated with reduced CD28 expression and increased cytotoxic potential. Functionally, these cells exhibit greater polyfunctionality than their CD57^−^ counterparts, with higher combined degranulation and cytokine production, particularly IFN-γ and TNF-α, following polyclonal stimulation. Importantly, CD57^+^ NKT-like cells display higher functional activity than their CD56^−^CD57^−^ counterparts, supporting the concept that, in this lineage, CD57 marks terminally differentiated yet functionally potent effectors rather than merely senescent cells [71].

### 3.2. CD4^+^ T Cells

In the CD4^+^ T-cell compartment, CD57 expression is likewise associated with distinct phenotypic and functional features, which differ from those observed in CD8^+^ T cells. In contrast, CD4^+^ T lymphocytes tend to lose CD27 and subsequently CD28 expression during differentiation [72,73]. Although, CD27lowCD57^+^ CD4^+^ T cells have also been identified [48]. CD57+ CD4+ T cells are functionally distinct from conventional helper T cells, as they display limited cooperation with B cells [74]. Nevertheless, upon stimulation, a subset of these CD57+ CD4+ T cells can upregulate CD40L (CD154), suggesting that they retain partial effector capacity despite their apparent senescent-like phenotype [55]. Activated CD57+ CD4+ T cells are cytotoxic and produce multiple cytokines, with higher functional responses than their CD57^−^ counterparts, supporting the concept that terminal differentiation in this subset is associated with a shift from proliferative capacity toward increased cytotoxic and secretory functions [35,55,75]. Specifically, CD57+ CD4+ T cells can produce a broad range of mediators, including IL-2 (low levels), IFN-γ, TNF-α, perforin, granzyme B and express multiple surface molecules associated with activation, migration, and innate-like responses. These include co-stimulatory molecules (OX40, 4-1 BB), chemokine receptors (CX3CR1, CCR5), Toll-like receptors (TLR2, TLR4), adhesion proteins (VLA-4, ICAM-1), and natural killer cell receptors such as NKG2C, NKG2D, CD11b and CD161 [55]. Together, these findings support the concept that CD57+ CD4+ T cells undergo a functional reprogramming characterized by reduced proliferative capacity but enhanced cytotoxic and secretory functions, positioning this subset as a key contributor to antiviral immunity and chronic inflammatory responses [76].

Thus, CD57 emerges as a robust marker of cytotoxic differentiation in T cells, more closely associated with cytotoxic effector programmes than alternative markers such as CD300a [35]. Consistently, CD57 expression is linked to functional specialization rather than uniform functional decline, as CD57^+^ T cells in both CD4^+^ and CD8^+^ lineages exhibit greater polyfunctional activity than CD57^−^ cells [76].

## 4. Are CD57^+^ T Cells Truly Senescent?

CD57 expression has long been employed as a phenotypic marker of senescence in human lymphocytes, particularly within the CD8^+^ T-cell compartment. Early seminal studies demonstrated that CD57+ CD8+ T cells exhibit hallmarks consistent with replicative senescence, including shortened telomeres, low telomerase activity, and severely impaired proliferative responses following T-cell receptor stimulation, while retaining the ability to produce effector cytokines [44]. Together with the frequent loss of costimulatory molecules such as CD27 and CD28, these features positioned CD57 as a core component of the senescent T-cell phenotype associated with ageing and chronic infection [77]. Thus, these cells have historically been interpreted as a characteristic feature of immunosenescence; however, these phenotypic changes more accurately reflect chronic antigen-driven differentiation [13]. Indeed, within the CD8^+^ TEMRA subset, CD57 expression distinguishes a terminally differentiated CD57^+^ population from a previously functionally uncharacterized CD57^−^ “younger” TEMRA population that exhibits high proliferative capacity and greater differentiation plasticity [78].

However, when CD57^+^ T cells are evaluated against the strict biological definition of cellular senescence, they do not consistently fulfil the required criteria. Cellular senescence is characterized by a combination of parameters, and no single marker is sufficient to establish it; rather, a cell can be reliably classified as senescent only when several of these features are detected simultaneously, including SA-β-galactosidase activity, stable proliferative arrest mediated by the activation of the tumour suppressors TP53 and CDKN2A/p16, and their downstream effectors CDKN1A/p21 and retinoblastoma-1 (RB1) family proteins, telomere attrition, accumulation of DNA damage, and signs of mitochondrial dysfunction [79,80]. Recent functional analyses further reinforce this reinterpretation by showing that CD27/28^−^CD57^+^ T cells do not meet core biological criteria of cellular senescence. These cells are not enriched for canonical senescence markers such as p16INK4A or senescence-associated β-galactosidase in either peripheral blood or tumour samples, and they retain measurable proliferative capacity in vitro, including the ability to divide in response to IL-2 or IL-15 stimulation. In parallel, they display preserved or enhanced effector activity, with increased expression of perforin, granzymes and CD107a, together with robust IFN-γ production. These data support the view that CD27/28^−^CD57^+^ T cells are better defined as highly differentiated effector-memory cells than as truly senescent lymphocytes [81]. Furthermore, in vivo labelling and longitudinal studies have demonstrated that CD57^+^ T cells can persist long-term and undergo intracompartmental proliferation, indicating that at least a fraction of these cells retains replicative potential in physiological settings [82]. Additionally, CD57^+^ T cells can display potent effector functions, including high cytotoxic capacity and robust production of IFN-γ and TNF-α, features that are not typical of classically senescent cells, which are generally characterized by functional decline [83,84,85,86].

Collectively, current evidence indicates that CD57^+^ T cells are not universally “truly senescent” in the strict biological sense. Rather, they represent a heterogeneous population strongly enriched for advanced differentiation, altered proliferative capacity, and features of immunological ageing, particularly within CD8^+^ compartments and CMV-experienced individuals (Table 1). While CD57 identifies lymphocytes with a high likelihood of replication incompetence and effector skewing, its expression alone is insufficient to define irreversible cellular senescence. Accurate interpretation, therefore, requires integrating additional phenotypic and molecular markers, including CD28, KLRG-1, PD-1, p16/p21 expression, and SA-β-gal activity, as well as consideration of clinical context and antigenic history [36]. This distinction is clinically relevant, as CD57^+^ cells may still actively contribute to immune responses and disease processes despite exhibiting senescence-associated traits. Different key markers of CD57^+^ T-Cell Subsets are described in Table 1. For conceptual clarity, the distinctions between cellular senescence, T-cell exhaustion, terminal differentiation, and immunological age are summarized in Table 2.

This table summarizes phenotypic markers, senescence-associated molecules, homing receptors, NK-like receptors, immune checkpoints, cytokines, and cytotoxic mediators reported in highly differentiated CD28null/CD57^+^ CD4^+^ and CD28null/CD57^+^ CD8^+^ T-cell subsets. Data are compiled from multiple published studies in chronic viral infection, immunosenescence, and inflammatory disease contexts. Primary supporting references: [45,48,55,75]. Arrows indicate: ↑, high expression; ↓ low/null expression.

**Table 2:** Conceptual distinction between senescence, exhaustion, terminal differentiation, and immunological age in lymphocytes.

Concept	Core Definition	Primary Driver	Proliferative Capacity	Effector Function	Typical Markers/Features	Reversibility
Cellular senescence	Stable cell-cycle arrest with senescence-associated molecular programme	Replicative stress, DNA damage, telomere attrition	Severely reduced or absent	Variable; often dysregulated secretory phenotype	p16INK4a ↑, p21 ↑, SA-β-gal^+^, DNA damage signals, telomere shortening	Generally irreversible
T-cell exhaustion	Functional hyporesponsiveness due to chronic antigen stimulation	Persistent antigen exposure (chronic infection, cancer)	Reduced	Decreased cytokine production and cytotoxicity	PD-1 ↑, TIM-3 ↑, LAG-3 ↑, TOX signature	Partially reversible (e.g., checkpoint blockade)
Terminal differentiation	Late-stage antigen-driven effector maturation	Repeated antigenic stimulation	Limited but not absent	Preserved or enhanced cytotoxicity	CD57^+^, CD28null, KLRG1^+^, high perforin/granzymes	Partially constrained but not fixed
Immunological age	Cumulative immune remodelling reflecting antigenic history rather than chronological age	Lifelong antigen exposure (especially CMV)	Subset-dependent	Often effector-skewed	Expansion of CD57^+^ and late-differentiated subsets, repertoire narrowing	Dynamic at system level

Arrows indicate: ↑, increase.

## 5. CD57 in CD4^+^CD8^+^ T Cells

Double-positive (DP) T cells account for 1–2% of circulating human T cells and are a heterogeneous population. They may display effector memory-like features, including the CD57 expression [87], suggesting antigen-experienced differentiation [88]. As demonstrated by Clénet et al., in healthy individual DP T cells express CD57 and show enhanced effector functions (INF-γ, CD107a, perforin production) [87]. These cells are distinguished by the lack of CCR7, CD27 and CD2 [89].

Consistent with the findings of Nascimbeni et al., DP T cells increase during viral infections (such as CMV, EBV and HIV). In these contexts, they demonstrate a more advanced differentiation profile compared to single-positive cells. Notably, the frequency of CD57 expression is higher in the DP subset than in CD4 single positive subset (28% vs. 7%), accompanied by the downregulation of CD28 and CD27 [90]. These data support the hypothesis that persistent antigenic stimulation drives the expansion toward a terminally differentiated, highly cytotoxic state.

## 6. CD57 in γδ T Cells

γδ T cells do not fully conform to “classical” immunosenescence trajectories as they retain robust effector competence with ageing [91,92]. Within this lineage, Vδ1^+^ cells exhibit a peripheral phenotype that more closely mirrors that of αβ CD8^+^ T cells during the combined influence of CMV and chronological ageing. In this context, CD57 expression aligns more with advanced differentiation and reduced proliferative potential, while cytotoxic capacity can remain preserved in defined subsets [91,93]. These observations indicate that some senescence-associated markers retain interpretations in Vδ1^+^ cells that resemble those in αβ T cells, albeit with functional nuances [94]. In contrast, Vδ2^+^ cells (typically Vγ9/Vδ2) deviate from these canonical patterns, as they are either less prone to cellular ageing or governed by mechanisms distinct from those of αβ T cells [91]. Subsequent studies converge on the view that Vδ2^+^ cells do not map neatly onto αβ-style differentiation markers (e.g., CD27, CD28) in terms of cytokine production and functional readouts [95,96]. Moreover, ligating KLRG1 on Vδ2^+^ cells does not reproduce the inhibitory effects observed in CD4^+^, CD8^+^, or NK cells, underscoring lineage-specific meanings for so-called senescence markers [97]. Taken together, these data support a model in which Vδ1^+^ cells exhibit age- and CMV-linked terminal differentiation reminiscent of αβ CD8^+^ T cells, whereas Vδ2^+^ cells display relative resistance to classical immunosenescence and maintain strong effector functionality despite the expression of markers like CD57 [92,98].

## 7. CD57 in NK Cells

The CD57 marker was originally identified on NK cells and was associated with natural killer activity. CD57 is variably expressed on different populations of NK cells. Both CD16^+^CD56dim cytotoxic NK cells and CD16^+^CD56bright inflammatory NK cells show a high expression of CD57. In contrast, NK cells with a regulatory profile CD16loCD56bright do not express CD57 [18]. Moreover, CD56^−^ NK cells expressing low levels of CD57, but not displaying canonical features of cellular senescence, have been reported in chronic viral infections, including HIV, hepatitis C virus, EBV and CMV [99].

CD57 is widely regarded as a marker of terminal differentiation in NK cells, with expression increasing along the maturation trajectory from CD56bright to CD56dim subsets and being associated with highly cytotoxic, functionally mature NK cells, whereas CD56bright NK cells typically show little or no CD57 expression [18]. The transition of NK cells from the CD56bright to the CD56dimCD16^+^ phenotype involves a marked decrease in NKp46, NKG2D, NKp30, and NKG2A expression, concomitant with the acquisition of CD16, and the expression of LIR-1 and KIR [100]. Specifically, the expression of CD57 in mature NK cells is closely related to the increased expression of markers NKG2C and CD8 [101]. Furthermore, CD57^+^NKG2Chigh NK cells have been proposed as a population of human CMV-specific “memory-like” NK cells [102,103,104], while polyfunctional CD8^+^ NK cells have been associated with slower disease progression in chronic HIV-1 infection [101]. Similarly to T cells, mature NK cells express CXCR1 and CX3CR1, which are chemokine receptors involved in the homing and migration of NK cells toward peripheral inflamed tissues, including the vascular endothelium. These receptors facilitate lymphocytic recruitment to sites of inflammation by responding to chemokine gradients, thereby enhancing their cytotoxic and immunoregulatory functions within inflamed or infected microenvironments. The expression of these homing receptors, together with the cell–cell adhesion function attributed to the CD57 marker, suggests that CD57^+^ NK cells, and by extension CD57^+^ T cells, play a local role in inflammatory processes. This combination of migratory and adhesive properties could facilitate the accumulation and effector activity of these cells within inflamed tissues [105].

Although CD57 expression in NK cells has traditionally been interpreted as a marker of terminal maturation rather than definitive cellular senescence, more recent frameworks propose that CD57 can contribute to the identification of a senescence-like NK phenotype when combined with additional features such as reduced proliferative capacity, impaired cytotoxicity, and senescence-associated signalling pathways. Importantly, CD57 alone shows limited specificity and is insufficient to define NK cell senescence in the absence of complementary molecular and functional criteria [106]. The gain of CD57 by CD56dim NK cells has been associated with a reduction in their proliferative capacity in response to pro-inflammatory cytokines, which could be associated with less expression of IL-2Rβ, IL-12Rβ and IL-18Rα receptors [18,37]. However, CD57^+^CD56dimCD16^+^ NK cells exhibit the same proliferative capacity as their CD57-counterparts, showing similar levels of the Ki-67 marker [37]. Additionally, CD57^+^CD56dimCD16^+^ NK cells have a marked lytic capacity related to their antibody-dependent cell-mediated cytotoxicity (ADCC) function with high levels of granzyme B, perforin, and degranulation markers such as CD107a [34].

Several studies have highlighted the role of CD57 expression in shaping the functional profile and adaptive-like features of NK cells under different physiological and pathological contexts. CD57 expression, often in combination with NKG2C, is particularly enriched in NK cells from CMV-seropositive individuals [107]. In healthy young CMV^+^ donors, an expansion of CD57^+^NKG2C^+^ NK cells has been described, which is associated with a more mature phenotype but also with a reduced ability to produce interferon-γ in response to heterologous antigens such as Bordetella pertussis and H1N1 influenza virus [108].

Similarly, in CMV^+^ kidney transplant recipients, dynamic changes in memory-like NK cell subsets have been observed. Pre-existing memory-like NK cells (NKG2C^+^CD57^+^FcεRγ^−^) tend to decrease over time, while pre-memory-like populations (NKG2C^+^CD57^+^FcεRγlow/dim) expand during periods of viremia and exhibit a stronger cytotoxic profile compared to non-viremic patients [109]. These findings suggest that viral reactivation and chronic antigenic stimulation drive phenotypic adaptations within the CD57^+^ NK compartment, promoting the development of memory-like subsets with enhanced effector capacity.

Recent data from COVID-19 convalescent individuals further support the link between CD57 expression and NK cell activation status. Higher frequencies of CD57^+^ NK cells have been reported in patients who experienced severe disease compared with mild or asymptomatic cases, indicating an association between CD57 expression and sustained NK activation [110]. Moreover, IFN-γ production in response to SARS-CoV-2 peptides has been correlated with the presence of CD57^+^NKG2C^+^ NK cells, although some individuals display robust responses even in their absence, suggesting heterogeneity in NK cell memory responses to viral infection. Given that CMV seropositivity is the major driver of CD57^+^NKG2C^+^ NK-cell expansion (37), the absence of CMV assessment in this study represents an important limitation. However, in other studies, increased proportions of NKG2ChiCD57^+^ NK cells have been observed predominantly in CMV-seropositive subjects, but not in CMV-seronegative ones, across several viral settings, including acute chikungunya [111], hantavirus infections [112], as well as chronic infections such as HIV-1 [113] and hepatitis B and C [114].

Altogether, these findings indicate that prior CMV exposure is a key requirement for the emergence of CD57^+^ NK-cell population, and CD57 definitively identifies a virus-driven, highly cytotoxic, terminally differentiated NK subset resulting from adaptive immune remodelling [115,116].

## 8. CD57^+^ Lymphocyte Expansion and Chronic Stimulation

CD57 expression across lymphocyte lineages is broadly associated with prolonged or repeated antigenic and cytokine-driven stimulation. In both T and NK compartments, the accumulation of CD57^+^ subsets is a recurrent feature of chronic immune activation and sustained environmental pressure, reflecting progressive differentiation and long-term immune adaptation. Rather than representing a lineage-specific phenomenon, CD57 upregulation emerges as a convergent immunophenotypic outcome of persistent stimulation, linking chronic exposure signals with stable shifts in lymphocyte subset composition and functional state.

It has been established that the central driver of CD57^+^ T-cell accumulation is chronic antigenic stimulation, particularly that imposed by persistent viral infections. Among these, CMV exerts a dominant and sustained impact on the T-cell compartment, promoting the expansion of highly differentiated CD8^+^ and CD4^+^ T-cell subsets characterized by CD57 expression, oligoclonal repertoire restriction, and reduced proliferative capacity. CMV-driven immune imprinting has been consistently associated with accelerated immune ageing, altered T-cell diversity, and systemic immune activation, positioning CMV as a key architect of the CD57^+^ T-cell landscape across the lifespan [8,13,35,55,75].

Importantly, the contribution of CMV to CD57^+^ T-cell expansion is frequently overlooked in studies examining CD57 expression in chronic diseases. This omission complicates the interpretation of “CD57-high” phenotypes, as CMV serostatus can profoundly shape the size, differentiation state, and functional profile of CD57^+^ T-cell populations independently of the primary disease under investigation. Recent high-dimensional immunophenotyping studies have reinforced the notion that CMV infection is a major confounder in analyses of late-differentiated T-cell subsets and must be systematically accounted for when attributing CD57^+^ T-cell expansion to disease-specific mechanisms [117,118].

Against this background, CD57^+^ T cells should be viewed not merely as markers of terminal differentiation, but as integrative indicators of cumulative immune history shaped by chronic antigenic exposure, viral coinfections, inflammatory signals, and host genetic factors. In the following sections, we review representative groups of representative pathological conditions, including major chronic viral infections, cardiovascular diseases, autoimmune diseases and cancer [119,120,121,122,123,124,125,126,127,128,129,130,131,132,133,134,135,136,137,138,139,140,141,142,143,144,145,146,147,148,149,150,151,152,153,154,155,156,157,158,159,160,161,162,163,164,165,166,167,168,169,170,171,172,173,174,175,176,177,178,179,180,181,182,183,184,185,186,187,188,189,190,191,192,193,194,195,196,197,198,199,200,201,202,203,204,205,206,207,208,209,210,211], in which CD57^+^ T cells have been implicated. We highlight their contribution to immune dysregulation and disease development, and emphasizing the need to interpret CD57 expression within the broader context of chronic stimulation and CMV-driven immune remodelling.

### 8.1. Major Chronic Viral Infections

**Figure 1:**
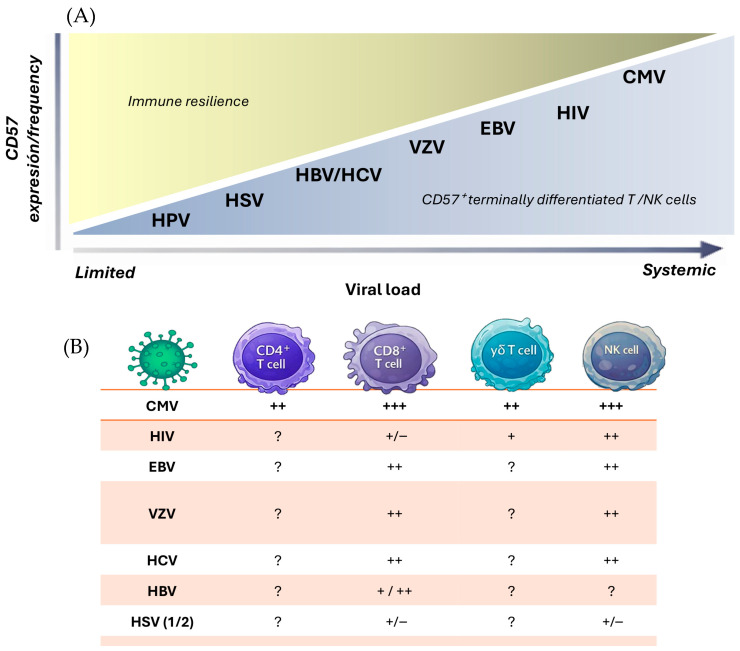
Chronic viral infections shape CD57 expression as a marker of cytotoxic differentiation along gradients of antigenic burden and immune compartment specialization. (**A**) Viruses are positioned along a continuum of viral load and systemic antigen exposure. Infections with limited or predominantly tissue-restricted persistence (e.g., HPV and HSV) show low or context-dependent CD57 imprinting, whereas viruses characterized by chronic stimulation or frequent reactivation (HBV/HCV, VZV, EBV, HIV, and especially CMV) are associated with progressive accumulation of CD57^+^ and CD57^++^ cytotoxic lymphocytes. This gradient reflects a shift from immune resilience toward terminal cytotoxic differentiation across T- and NK-cell compartments. (**B**) CD57 imprinting is lineage-dependent. CD8^+^ T cells show the strongest enrichment in CMV (CD57^+++^), with intermediate accumulation in EBV, VZV, and HCV, and more heterogeneous patterns in HIV and HSV. CD57^+^ cytotoxic CD4^+^ T-cell expansion is strongly CMV-associated; reports in other chronic infections lack CMV stratification and remain inconclusive. γδ T cells can acquire CD57 under chronic viral stimulation—most clearly described in CMV—where it marks clonally expanded, cytotoxic, innate-like populations rather than uniform exhaustion. In NK cells, CMV characteristically drives the emergence of adaptive CD57^++^ NK subsets, whereas other viral infections produce milder, heterogeneous, or tissue-restricted CD57 remodelling. Note: These CD57 scores are literature-based and come from heterogeneous cohorts; many studies did not control for CMV serostatus. Because CMV strongly drives CD57^+^ differentiation, part of the signal in non-CMV infections may be CMV-related, so interpretation should be cautious. Scoring shown in the table: +++ high, ++ moderate, + low, +/− variable, ? insufficient evidence.

Chronic CMV infection is widely supported as a major—and in many cohorts quantitatively predominant—driver of CD57 acquisition and late T-cell differentiation. However, this process is multifactorial and also influenced by chronological age, inflammatory burden, metabolic status, and co-infections. Comparative cohort studies indicate that CMV serostatus often explains a larger proportion of the variance in CD28null and CD57^+^ T-cell expansion than age alone, particularly within CD4^+^ subsets [140], whereas CD8^+^ compartments show a more mixed contribution of CMV- and age-related effects. Expansions of CD57^+^ lymphocytes—predominantly within the CD8^+^ compartment—have also been documented in association with other persistent viral infections, including human immunodeficiency virus (HIV), Epstein–Barr virus (EBV), hepatitis C virus (HCV), varicella–zoster virus (VZV), and herpes simplex virus (HSV) (Table 3, Figure 1) [119,120,121,122,123,124,125,126,127,128,129,130,131,132].

Furthermore, recent cohort-level analyses indicate substantial interindividual heterogeneity in the degree of CMV-associated memory skewing, with marked global compartment distortion occurring only in a subset of CMV-seropositive individuals. This variability suggests that host factors and additional inflammatory or environmental influences modulate the magnitude of CMV-driven immune remodelling [133].

In older adults, these CD28null/CD57^+^ CD8^+^ T-cell populations are often enriched for CMV- and EBV-specific responses, indicating that lifelong persistent viral exposure—more than chronological ageing alone—drives oligoclonal accumulation of highly differentiated CD8^+^ T cells [45,48]. Functionally, CD28nullCD57^+^ CD8^+^ T cells show high granzyme and perforin expression supporting antiviral cytotoxicity and immune surveillance, as it has been shown in the context of CMV infection and HIV–CMV co-infection, where CD28nullCD57^+^CX3CR1^+^ memory subsets are linked to stable viral control [134,135,136]. However, persistent oligoclonal expansion of these late-differentiated cells may restrict immune repertoire diversity, reduce space for naïve and central memory T cells, and impair responses to new antigens and vaccination. In this sense, CD28null CD8^+^ subsets, especially CD57^+^ or senescent/regulatory populations, have also been associated with suppressive activity and immunosenescent profiles linked to poorer outcomes in ageing and malignancy [49,137,138]. Overall, chronic viral infections act as major contributors to CD8^+^ T-cell remodelling, with ageing often amplifying infection-driven effects [13,55,76,77].

In contrast, CD57-associated differentiation is less consistently observed in the CD4^+^ compartment. Although virus-specific CD4^+^ T cells are frequently detectable across chronic viral infections, they are not uniformly linked to CD57 expression nor to the same degree of terminal differentiation seen in CD8^+^ cells. Current evidence indicates that the emergence and accumulation of CD28nullCD57^+^ CD4^+^ T cells is predominantly driven by CMV infection. Quantitative cohort analyses show that age-related increases in these subsets are largely restricted to CMV-seropositive individuals, whereas little or no expansion is observed with ageing alone in CMV-seronegative subjects [11,139,140]. When present, CD57+ CD4+ T cells represent late-differentiated effector populations with reduced proliferative capacity but preserved cytokine and cytotoxic potential.

Similarly, in NK cells, CD57 expression is a hallmark of terminal differentiation which is also strongly influenced by chronic viral exposure. Once more, CMV is the best-established and often dominant determinant of CD57^+^ NK-cell expansion and adaptive-like NK phenotypes. Although infections such as HIV, EBV, and chronic hepatitis are associated with increased frequencies of differentiated NK cells, studies incorporating CMV serology and CMV-associated phenotypic signatures show that these subsets largely correspond to canonical CMV-driven adaptive NK populations [141,142,143]. In co-infection settings, non-CMV viruses tend to modify activation state, tissue distribution, or clinical associations of CD57^+^ NK cells rather than generate these populations de novo [144]. Without CMV stratification, attribution of CD57^+^ NK expansion to other viruses remains unreliable.

### 8.2. Cardiovascular Diseases

Several studies have observed an expansion of CD57^+^ T lymphocytes in patients with cardiovascular disease. Coronary artery disease (CAD) was associated with an increase in CD57+ CD8+ T and CD28nullCD8^+^ T cells in 29 of 43 (67%) CMV-seropositive patients younger than 60 years. Multiple regression analysis showed that CAD per se is a determinant of alterations in T lymphocyte subpopulations. Authors suggest that the expansion of these subpopulations may be stimulated by persistent antigens associated with CMV infection found in cardiovascular tissue, thus exacerbating the inflammatory response [145].

A high frequency of CD57+ CD8+ T cells has also been found in patients with myocardial infarction between 60 and 80 years of age [15,146,147]. These subpopulations were associated with cardiovascular mortality 6 months after the cardiovascular event. Furthermore, CD57+ CD8+ T cells showed a higher cytotoxic profile, releasing perforin and granzymes A and B, compared to their CD57^−^CD8^+^ counterparts [145]. The CD28nullCD57^+^ CD4^+^ T subpopulations found in cardiovascular diseases are more homogeneous, showing mainly pro-inflammatory characteristics. In patients with unstable angina pectoris, an increase in CD28nullINFγ^+^ CD4^+^ T lymphocytes was observed compared to patients with stable angina pectoris and healthy patients [148]. Furthermore, HIV+ patients and CMV coinfections showed a high expansion of memory CD57+ CD4+ T cells in the atherosclerosis plaque. These cells exhibited overexpression of the vascular endothelial receptor CXBCR1 and CD2. Furthermore, the presence of IL-15 stimulated the migration of these cells, associated with a release of TNFα, granzyme B and perforin. Consequently, it can be thought that CMV infection enhances the expansion of CD57+ CD4+ T cells, this being a key factor for the development of vascular damage in immunocompromised individuals [143]. The expansion of CD28nullCD4^+^ T cells has been observed in patients with a risk factor for developing atherosclerosis and patients with acute coronary syndrome aged between 40 and 80 years. Upon anti-CD3 stimulation these cells produced high levels of perforin, granzyme A and B, and TNFα [149]. Moreover, it has been demonstrated that CD28nullCD4^+^ T cells are associated with post-operative atrial fibrillation and higher levels of CRP [150].

In CAD, the occurrence of increased frequencies of CD28nullCD57^+^ CD8^+^ T cells has also been shown. Importantly, both CMV seropositivity and coronary artery disease were independently linked to this expansion, supporting the idea that chronic viral antigen exposure and CAD-associated inflammation act together to promote immunosenescence [145]. A recent study from our group further confirmed the involvement of CD57^+^ cells in isolated CAD (iCAD) and CAD associated with aortic stenosis (ASCAD), highlighting their potential utility as biomarkers for identifying individuals at increased risk and for monitoring disease progression [151]. Distinct immunological profiles were identified between iCAD and ASCAD. iCAD was characterized by enhanced immune activation, evidenced by increased inflammatory CD14^+^CD16^+^ monocytes, elevated Treg frequencies, and greater differentiation of CD4^+^ T cells toward effector memory (TEM) and terminally differentiated (TEMRA) phenotypes. Conversely, ASCAD was associated with marked immunosenescence, reflected by increased neutrophil counts, lymphopenia, and heightened cytotoxic activity of NK and T cells. The predictive model accurately discriminated between iCAD and ASCAD, highlighting CD4^+^ T cell memory subsets and CD57 expression as key distinguishing markers. Overall, the findings indicate that iCAD is primarily driven by immune activation, whereas ASCAD is dominated by immunosenescence and cytotoxicity.

In chronic heart failure (CHF), the increase in the frequency of CD28null T lymphocytes was studied in young and elderly individuals. The frequency of CD45^+/−^ CD28null CD4^+^ T cells in both age groups was related to CMV seropositivity and to pathology. In contrast, the frequency of CD45^+/−^CD28null CD8^+^ T cells was only associated with CMV seropositivity. Furthermore, the CD4^+^ subpopulation showed higher expression of the CD69 activation receptor than the CD8^+^ subset. These data suggested that the differentiation of these populations contributed to the exacerbation of the disease [152].

The presence of these expansions in the context of cardiovascular disease is a relevant indicator for predicting and estimating vascular damage in individuals with risk factors. However, few studies account for CMV seropositivity in the target population, even though it may alter T lymphocyte subpopulations regardless of age. Therefore, further research is needed to clarify the causal relationship between these cardiovascular events and underlying immunopathological mechanisms. Such evidence would support the development of effective combination therapies aimed at preventing or slowing cardiovascular damage.

### 8.3. Autoimmune Diseases

Several studies have evaluated the expression of CD57 on T lymphocytes across a wide range of autoimmune diseases, supporting the concept that CD57^+^ T cells represent a recurrent immunological feature in chronic autoimmune inflammation. Table 4 summarizes some autoimmune diseases with reported increased frequencies of CD57^+^ T cells, predominantly within the CD8^+^ compartment but also affecting CD4^+^ subsets. In many of these conditions, CD57^+^ T cells display phenotypic and functional characteristics consistent with terminal differentiation, clonal expansion, and enhanced cytotoxic or pro-inflammatory capacity, suggesting a potential pathogenic role in tissue damage and disease progression.

In rheumatoid arthritis, the disease with the most extensive evidence, CD57^+^ T cells have been described both in peripheral blood and inflamed tissues, particularly synovial fluid and bone marrow [153,154,155,156,157]. These cells often exhibit restricted TCR repertoires, high interferon-γ production, and associations with disease activity or duration, indicating antigen-driven expansion and functional relevance in local inflammation. Similar observations of tissue-enriched CD57^+^ cytotoxic T cells have been reported in multiple sclerosis meningeal infiltrates [158], systemic sclerosis–associated lung fibrosis [159], Crohn’s disease intestinal lesions [153,154,155,156,157,158,159,160], and alopecia areata [161,162]; among others, reinforcing the notion that CD57 expression marks chronically stimulated effector T cells involved in organ-specific autoimmunity

Despite the strong biological overlap between CD57 expression, T-cell senescence, and chronic viral exposure, very few studies summarized in Table 4 explicitly evaluated CMV serostatus. Only early work in rheumatoid arthritis [153], and selected analyses in type 1 diabetes [163] and celiac disease [164] incorporated CMV-related assessments, while the vast majority of studies interpreted CD57^+^ T-cell expansions solely within an autoimmune framework. This represents a critical limitation, as CMV infection is a major driver of CD57+ CD8+ T-cell accumulation and clonal expansion in the general population. Failure to account for CMV seropositivity may therefore confound the attribution of CD57^+^ T-cell expansions to autoimmune mechanisms alone. Consequently, incorporating systematic CMV evaluation in future studies will be essential to disentangle virus-driven immune remodelling from disease-specific autoimmune processes and to better define the true pathogenic significance of CD57^+^ T cells in autoimmunity.

### 8.4. Cancer

The type of cancer and its stage are characteristics that can affect lymphocyte expansion. T lymphocyte subpopulations could be immunomodulatory or cytotoxic, favouring or combating tumour cell growth. In individuals suffering from gastric cancer, CD57+ CD8+ T lymphocytes have been found to expand only in patients with advanced-stage disease. In addition, a negative correlation was established between the production of IFNγ and that of CD57^+^ T cells. Specifically, it was observed that in patients with advanced-stage disease, the frequency of CD57^+^perforin^+^ T cells was lower. This suggests that this cell population could be ineffective in antitumor defence, favouring a tolerogenic state that allows uncontrolled growth of tumour cells [165].

In another type of cancer, such as melanoma, patients with less than 23% CD57^+^CD8high T cells before treatment survived longer than patients with more than 23% of this population, suggesting an immunosuppressive activity in these CD8^+^ T lymphocytes [166]. Similar results were obtained in patients with small cell lung cancer, where patients with a better response to chemotherapy showed less than 20% CD57^+^CD8high T cells, less than 20% and less than 3% FOXP3^+^ cells [167].

Other authors have also shown that patients with stage IV carcinoma with high percentages of CD57+ CD8+ T cells had a prolonged, progression-free survival (Progression-Free Survival, PFS) [68], greater than patients with poorly differentiated CD8^+^ T cells. In patients with clear cell renal carcinoma, CD57^+^ T cell density was negatively related to some parameters such as grade, disease state (pT), and metastasis. This relation raises a possible antitumor role of CD57^+^ T lymphocytes [168].

The controversy between antitumor and immunomodulatory activity in various types of cancer is due to the versatility of CD57+ CD8+ T lymphocytes. Some subpopulations could be highly cytotoxic and destroy tumour cells, while others would promote peripheral tolerance, thus allowing continued tumour growth. We could say that leukocyte markers such as CD57, FOXP3 and other NK cell receptors NKG2D, NKG2A, KIRs (killer-cell immunoglobulin-like receptors), CD16 (FcγRIII), and DNAM-1 (CD226) provide significant data on the immune status of the patient, to predict the prognosis of the disease.

In bone marrow and peripheral blood of patients with plasma cell dyscrasia (PCD), CD57+ CD8+ T cells also have high expression of antigen 1 associated with lymphocyte function (LFA-1) and CD57. This phenotype was associated with an inhibition of the antigen-specific T cell response, compared to healthy donors [169].

Beyond the CD8^+^ compartment, multiple studies indicate that CD57+ CD4+ T cells also expand in several cancer settings, although their frequency, localization, and functional significance appear to be context-dependent. In solid tumours such as breast cancer, accumulation of highly differentiated KLRG1^+^CD57^+^ CD4^+^ T cells has been described in peripheral blood, tumour tissue, and tumour-draining lymph nodes, where these cells display a functionally specialized phenotype within the tumour immune microenvironment. In this study these cells were associated with improved overall survival, highlighting the potential value of monitoring these subsets as prognostic biomarkers during patient follow-up [73]. Similar enrichment of differentiated CD57+ CD4+ subsets has also been reported in hematological malignancies, where CD57 expression marks late-stage differentiated CD4^+^ T cells with reduced proliferative capacity and altered helper function, contributing to impaired antigen-specific responses in disorders such as plasma cell dyscrasias and related B-cell malignancies. In a cohort of 82 patients with follicular lymphoma, four immune subgroups were identified with distinct T-cell profiles. Higher levels of early-differentiated T cells were linked to better survival, while late-differentiated T cells, especially CD57^+^ TFH cells, were associated with early disease progression and poorer survival. Single-cell analysis showed CD57^+^ TFH cells have inflammatory, exhausted, and apoptosis-prone gene signatures [170]. Another study reports that patients with glioblastoma multiforme, particularly those with HCMV-positive tumours, exhibit systemic immunosuppression together with an expansion of highly differentiated CD4^+^ T-cell subsets, including CD28nullCD4^+^ and CD57+ CD4+ populations. Increased frequencies of these senescence-associated CD4^+^ T cells were linked to reduced overall survival, supporting an association between virus-driven immune ageing profiles and poor clinical outcome [171].

## 9. Clinical Implications and Therapeutic Perspectives

From a clinical standpoint, the implications of CD57 expression in chronic infection have been addressed elsewhere and can be summarized briefly: although CMV is the main and quantitatively dominant driver of CD57-associated differentiation, other chronic infections can also modulate CD57 expression by altering inflammatory tone, tissue trafficking, and immune homeostasis. Nevertheless, the key interpretive challenge is that CMV often defines the baseline differentiation landscape on which other infections act, making CMV stratification essential for accurate clinical inference [141,142].

Importantly, CD57 itself should not be interpreted as a direct therapeutic target. Rather than representing a druggable molecule or a cell-depletion marker, CD57 primarily functions as a phenotypic indicator of cumulative antigenic stimulation and late-stage immune differentiation. Accordingly, current therapeutic strategies are more realistically directed at upstream drivers of CD57^+^ cell expansion—such as chronic viral activity, inflammatory signalling, and immune dysregulation—rather than at CD57-expressing cells per se. NK and CD8^+^ T cells expressing CD57 are often expanded in chronic infection and inflammation, reflecting cumulative antigenic pressure rather than irreversible dysfunction. While these cells retain strong cytotoxic potential, their terminal differentiation state is associated with reduced proliferative capacity and diminished adaptability to new immune challenges. Therapeutic strategies that broadly modulate immune activation, such as antiviral treatment of chronic infections, anti-inflammatory interventions, or immune checkpoint modulation, may therefore influence CD57-associated phenotypes indirectly by altering CMV reactivation dynamics rather than by directly targeting CD57^+^ cells themselves. This is particularly relevant in settings where CMV is not clinically apparent but immunologically active [172]. Regarding vaccine responses, it represents one of the most important translational contexts in which these concepts converge. CMV-associated immune remodelling, characterized by expansion of CD57^+^ late-differentiated T cells and contraction of naïve T-cell pools, has been repeatedly linked to impaired responses to vaccination, especially in older adults and chronically infected populations. Seminal studies have shown that CMV seropositivity is associated with altered cellular and humoral responses to influenza vaccination, independent of chronological age [173,174,175]. In this framework, reduced vaccine efficacy observed in chronic hepatitis, HIV infection, or other long-standing inflammatory conditions may partially reflect CMV-driven immune differentiation rather than pathogen-specific immune defects.

Other chronic infections can exacerbate CMV-associated limitations on vaccine responsiveness by increasing inflammation or altering lymphocyte trafficking. Without CMV stratification, reduced vaccine-induced immunity may be misattributed to disease-specific effects rather than reflecting differences in the magnitude of an underlying CMV-imprinted immune state, with important consequences for vaccine trial interpretation and consistency. From a therapeutic perspective, these findings suggest that CMV-targeted interventions, through antivirals, immunomodulation, or future CMV vaccines, may enhance immune repertoire diversity and improve responses to heterologous vaccines and immunotherapies, supporting the integration of CMV biology into therapeutic and vaccinology frameworks.

In summary, although multiple chronic infections can influence CD57 expression, CMV remains the most influential and best-characterized driver. Therapeutic and vaccine-related outcomes are therefore best interpreted when CD57 is viewed as a composite marker reflecting CMV history modified by additional infectious and inflammatory pressures, rather than as a pathogen-specific indicator in isolation [141,173].

## 10. Conclusions and Future Directions

CD57 is a valuable but context-dependent marker of immune differentiation in chronic viral infection, reflecting cumulative antigenic exposure rather than uniform dysfunction. Across infections, CMV is the main and best-characterized driver of CD57-associated phenotypes in NK and T cells, while other chronic viruses mainly influence their magnitude and clinical impact. Clinically, this hierarchy is critical for biomarker interpretation and supports CMV stratification to avoid misattributing immune ageing, exhaustion, or disease severity to the infection under study. Therapeutically, CD57^+^ populations are best viewed as functionally competent but terminally differentiated—cytotoxic yet proliferation-limited—favouring strategies that restore immune balance rather than broadly depleting differentiated cells. Integrating CMV biology into therapeutic and vaccine frameworks may improve immune resilience and immunotherapy responses, especially in ageing and high-risk groups. Longitudinal and interventional studies will be needed to define when CD57 reflects adaptive specialization versus maladaptive constraint and to guide precise immune stratification and modulation

## Figures and Tables

**Table 1:** Characteristics of CD57^+^ T-Cell Subsets.

Marker Group	Typical Change	CD28null/CD57^+^ CD4^+^ T Cells	CD28null/CD57^+^ CD8^+^ T Cells
Costimulatory receptors	Loss	CD28 ↓	CD28 ↓
Terminal differentiation	Increase	CD57 ↑	CD57 ↑
Cell cycle/senescence	Increase	p16, p21, p53 ↑	p16, p21, p53 ↑
Survival/apoptosis	Altered	Bcl-2 ↓/variable, CD95 ↑	Bcl-2 ↓/variable, CD95 ↑
Homing receptors	Decrease	CCR7 ↓, CD62L ↓	CCR7 ↓, CD62L ↓
Inflammatory trafficking	Increase	CCR5 ↑, CX3CR1 ↑	CCR5 ↑, CX3CR1 ↑
Differentiation state	Shift	Effector/cytotoxic CD4	TEMRA/effector
NK-like receptors	Increase	KLRG1, KIR, NKG2D ↑	KLRG1, KIR, NKG2D ↑
NK-associated markers	Subset increase	CD16^±^, CD56^±^	CD16^+^, CD56^+^ subsets
Adhesion molecules	Increase	LFA-1 ↑, VLA-4 ↑	LFA-1 ↑, VLA-4 ↑
Checkpoint receptors	Variable ↑	PD-1, CTLA-4^±^	PD-1, CTLA-4^±^
Regulatory markers	Subset	CD39^±^, FOXP3 rare	CD39^±^
Cytokines	Increase	IFN-γ ↑, TNF-α ↑	IFN-γ ↑, TNF-α ↑
Cytotoxic mediators	Increase	Granzyme B ↑, Perforin ↑	Granzyme B ↑, Perforin ↑
Degranulation	Increase	CD107a ↑	CD107a ↑

**Table 3:** Summary of CD57 findings in Major Chronic Viral Infections and CD57 Expression and CMV evaluation.

Infections	Role of CD57 in Immune Response	CMV	Refs.
HIV	CD57 marks antigen-driven T-cell differentiation and immune ageing under chronic stimulation and ART.	No	[44]
	CD57+ CD8+ T cells: high cytotoxicity with impaired proliferation and replicative senescence.	No	[44]
	CD8^+^ dysfunction can develop independently of CD57 or classical exhaustion markers.	No	[176]
	CD57+ CD8+ patterns strongly shaped by CMV; treated HIV shows CD28nullCD57^−^ skewing.	Yes	[177]
	CD28nullCD57^+^ CD8^+^ T cells associate with subclinical cardiovascular disease.	No	[178]
	CD8^+^CD57^+^ T cells correlate with broader HIV neutralization in viremic controllers.	No	[121,179]
	CD28nullCD57^bright^ CD4^+^ T cells retain IFN-γ effector activity and contribute to persistent activation.	Yes	[180]
	CD57 marks cytotoxic CD4^+^ T cells enriched in granzyme/perforin and T-bet/Eomes programmes.	No	[181]
	CMV drives CD57^+^CX3CR1^+^ cytotoxic CD4^+^ T cells with vascular-homing phenotype.	Yes	[182]
	CD57^+^/NKG2C^+^ adaptive NK expansion is enriched in CMV^+^ individuals.	Yes	[183,184]
	CD57^+^ γδ T cells show a differentiated phenotype; Vδ2^+^ CD57 associates with later neutralizing breadth.	No	[122]
EBV	CD57 marks differentiated NK and CD8^+^ T cells supporting long-term cytotoxic immune surveillance.	No	[185]
	Acute EBV expands differentiated NK cells, including CD57^+^ subsets that persist into latency.	No	[186]
	EBV-specific CD8^+^ T cells acquire CD57 with repeated antigen exposure and retain cytotoxic function	No	[158]
HCV	Chronic HCV drives CD57^+^ late differentiation across T and NK compartments.	No	[187]
	CD28nullCD57^+^ HCV core–specific T cells correlate with fibrosis severity.	No	[188]
	CD57 enrichment reflects reduced proliferative reserve with preserved cytotoxicity.	No	[189]
	MAIT cells show CD57 enrichment with depletion and exhaustion phenotype.	No	[190]
	NK repertoire remodelling persists after DAA clearance.	Yes	[191]
	DAA partially restores NK function but not full differentiation structure.	No	[192,193]
	NK receptor/effector imbalance contributes to chronic inflammation.	No	[194]
	Altered CD57+ CD4+ T-cell frequencies reported in HCV-associated HCC.	No	[195]
HSV	CD57 marks late-differentiated HSV-specific CD8^+^ T cells shaped by repeated reactivation.	No	[196,197]
	CD57^+^ HSV-specific CD8^+^ T cells enriched in symptomatic disease with exhaustion-like phenotype.	No	[198,199]
	NK ADCC pathways and host Fc genetics influence HSV control.	No	[200,201,202]
	Recurrent HSV-2 does not drive systemic expansion of CD57^+^ differentiated NK subsets. CMV serostatus is a major determinant of NK CD57 patterns in HSV studies.	Yes	[18,203]
VZV	CD57 marks late-differentiated cytotoxic T cells associated with age-related decline in VZV cellular immunity.	No	[204]
	In older adults, CD57^+^PD-1^+^ VZV-specific T cells associate with poor vaccine expansion and recall responses	No	[205]
	VZV-specific T cells are enriched in skin with durable tissue-resident imprinting after zoster.	No	[206,207]
	VZV can infect NK cells and induce CD57 acquisition with reduced CD16 and altered homing phenotype.	No	[208]
	Ganglia show relatively low VZV-specific CD8^+^ T-cell prevalence vs. HSV, suggesting limited CD8^+^ ganglionic control.	No	[209]

Abbreviations: CD, cluster of differentiation; CMV, cytomegalovirus; HIV, human immunodeficiency virus; EBV, Epstein–Barr virus; HCV, hepatitis C virus; HSV, herpes simplex virus; VZV, varicella–zoster virus; HCC, hepatocellular carcinoma; NK, natural killer; MAIT, mucosal-associated invariant T; γδ T cells, gamma delta T cells; DAA, direct-acting antivirals; ART, antiretroviral therapy; ADCC, antibody-dependent cellular cytotoxicity; CX3CR1, C-X3-C motif chemokine receptor 1; KIR, killer-cell immunoglobulin-like receptor; NKG2A/NKG2C, natural killer group 2A/2C receptors; PD-1, programmed cell death protein 1.

**Table 4:** Main findings on CD57^+^ T cells in representative autoimmune diseases and CMV evaluation.

Disease	Findings on Cd57^+^ T Cells	CMV	Ref.
Rheumatoid arthritis (RA)	RA patients show expanded CD57+ CD8+ T cells with restricted TCR Vβ5^+^/Vβ13^+^ repertoires, consistent with antigen-driven clonal expansion and a potential pathogenic role	YES	[153]
	Expanded, activated CD57^+^CD3^+^ lymphocytes in RA synovial fluid contribute to local inflammation.	NO	[154]
	Expanded CD57+ CD8+ T cells in blood and joint compartments associate with disease duration.	NO	[155]
	CD57+ CD4+ T cells in RA are associated with disease severity and high IFN-γ output.	NO	[156]
	Interleukin-15 selectively expands CD28nullCD57+ CD4+ T cells, which are increased in active RA	NO	[157]
Multiple sclerosis	CD57+ CD8+ T-cell enrichment in meningeal infiltrates associates with MS pathology	NO	[158]
Systemic lupus erythematosus	CD57+ CD4+ senescent T cells were key contributors to the immunopathogenesis of LES	NO	[210]
Systemic sclerosis	CD57^+^ cytotoxic CD8^+^ T cells play a central role in lung injury in fibrotic lung disease in systemic sclerosis.	NO	[159]
Psoriasis	CD57+ CD4+ and CD8^+^ T cells are enriched in unaffected vs. lesional psoriatic skin	NO	[211]
Type 1 diabetes	Memory CD57+ CD8+ T-cell subsets associate with C-peptide measures in young children.	YES	[163]
Alopecia Areata	Lesional skin is enriched in CD57^+^CD3^+^CD8^+^ lymphocytes driving cytotoxic inflammation	NO	[161]
	Multiple CD56^bright^ NK cell subsets, including CD8^+^, CD57^+^, and CD38^+^ phenotypes, are expanded in AA	NO	[162]
Inflammatory bowel disease	NK-like cytotoxic CD57+ CD8+ TEMRA cells are enriched in inflamed Crohn’s tissue	NO	[160]
Celiac disease (CD)	Subclinical celiac disease shows CD57^+^ T-cell expansion independent of CMV, with reduced Vδ1^+^ T cells and stable NK/NKT subsets.	Yes	[164]

## Data Availability

No new data were created or analyzed in this study.
